# Epidemiologic assessment of benzodiazepine exposure among suicide deaths in Colorado, 2015–2017

**DOI:** 10.1186/s12889-020-09250-y

**Published:** 2020-07-22

**Authors:** Tista Ghosh, Kirk Bol, Maria Butler, Barbara Gabella, Andrea Kingcade, Gabriel Kaplan, Lindsey Myers

**Affiliations:** Colorado Department of Public Health and Environment, 4300 Cherry Creek Drive South, Denver, CO 80246 USA

**Keywords:** Benzodiazepines, Suicide, Colorado

## Abstract

**Background:**

Suicide rates have been climbing in the U.S., particularly in Rocky Mountain states such as Colorado. Benzodiazepines have been linked with suicidal ideation, but there have been few population level assessments of this link. We conducted a public health assessment to determine the epidemiology and prevalence of recent benzodiazepine exposure, among suicide deaths in Colorado from 2015 to 17.

**Methods:**

This epidemiologic assessment linked Colorado’s Prescription Drug Monitoring Program, death certificate data, and Violent Death Reporting System to determine patterns of benzodiazepine exposure among suicide deaths in Colorado between 2015 and 2017. Recent benzodiazepine exposure was defined as receiving a prescription within 30 days of death or having a positive toxicology screen post-mortem.

**Results:**

Among the 3465 suicide deaths in Colorado between 2015 and 2017, 20% had recent benzodiazepine exposure, and nearly 50% of those also had recent opioid exposure. Recent benzodiazepine exposure was more common among females than males (34% versus 16%). Among suicide deaths, those who died via drug overdose were more likely to have had recent benzodiazepine exposure (48%), compared to suicides by firearm (17%), hanging/asphyxiation (13%) and all other methods (approximately 20%).

**Conclusions:**

Benzodiazepines have been linked to suicidal ideation, but population level assessments of benzodiazepine exposure among suicide deaths are rare. Our epidemiologic assessment indicates a relatively high prevalence of recent benzodiazepine exposure that warrants further investigation from both clinical and public health perspectives.

## Background

Benzodiazepines are a class of psychoactive drugs that are commonly prescribed for conditions such as anxiety, muscle spasms, seizures, insomnia, and alcohol withdrawal. In 2017, more than 1.5 million benzodiazepines prescriptions were dispensed in Colorado, the second most common class of controlled substances, according to the Colorado Prescription Drug Monitoring Program [1]. Benzodiazepine use is common in Colorado, the U.S., and in other countries, though how use is measured and reported varies in the literature, and use is more common in older ages and women [2–7]. In 2008, 5.2% of U.S. adults ages 18–80 years old filled a benzodiazepine prescription [3]. A more recent estimate is that 12.6% of U.S. adults ages 18 years or older used a benzodiazepine in the last year or 10.4% used them as prescribed, based on a different data source [8]. High rates of benzodiazepine use, especially long-term use in older adults, might reflect limited access to effective alternatives and/or patient preference [3, 9]. Benzodiazepines are federally-controlled substances, since long term use has been shown to lead to physical dependence. In addition, recent literature reviews suggest that benzodiazepines may have another negative side effect: suicidal ideation [10, 11]. However, there has been little study of the role of benzodiazepines in suicide from a public health perspective.

Suicide is a public health crisis in Colorado, resulting in 1271 suicide deaths in 2017. Suicide is consistently a leading cause of death in the state. In 2016, suicide rates increased 25.4% nationwide since 1999, and are even higher in the Rocky Mountain states, such as Colorado [12]. In Colorado, the age-adjusted annual rate of suicide was 23.2 deaths per 100,000 persons (2014–2016), a 34.1% increase from 1999 to 2001 [12]. In comparison, the U.S. rate was 15.4 deaths per 100,000 population (2014–2016) [12]. Numerous risk factors for suicide, ranging from gender to occupation to mental healthcare access to altitude, have been examined [13–15]. However, few analyses have been conducted to assess whether benzodiazepines play a role in suicide at the population level. The Colorado state health department therefore examined the prevalence and patterns of benzodiazepine exposure among suicide deaths, to determine whether further investigation into the role of benzodiazepines is necessary from a population health standpoint. This study was the first of its kind to use two common state public health databases in the U.S.: prescription drug monitoring program and violent death reporting system, which have similar counterparts in other countries.

## Methods

State-employed toxicology staff conducted a literature review to assess biologic plausibility of benzodiazepine use and suicidal ideation, in response to citizen inquiry and concern. This review suggested an increased risk of suicide while on benzodiazepines or when dosing is abruptly stopped or tapered [16–20]. State-employed epidemiologists then examined patterns of benzodiazepine use among suicide deaths by linking Colorado’s Prescription Drug Monitoring Program (PDMP), an electronic database that tracks the dispensing of controlled substances, to the Colorado Violent Death Reporting System that contains information from coroners’ and medical examiner’s records, law enforcement reports, and death certificate data [21, 22].

We defined the study population as suicide deaths occurring in Colorado between 2015 and 2017, identified using Colorado’s death certificate registry based on suicide as the underlying cause of death (ICD-10 codes X60-X84 and Y87.0). We subsequently linked these suicide deaths to the Colorado PDMP, comprising approximately 42 milliion records of controlled substances prescriptions dispensed between 2014 and 2018. This time period represents both the earliest and latest complete year of PDMP data available, which in turn afforded a minimum one-year retrospective of benzodiazepine prescriptions filled prior to death. These suicide deaths were additionally linked to records from the Colorado Violent Death Reporting System from 2015 to 2017, representing the latest complete years of enhanced data concerning toxicology status, including benzodiazepine exposure, at the time of death. We identified three subgroups of suicide deaths: (1) “ever dispensed a benzodiazepine,” defined as having received a prescription for a benzodiazepine ever during the timeframe of the look-back at the PDMP data (2014–2017); (2) “recent benzodiazepine exposure,” defined as benzodiazepines prescribed within 30 days of death or a positive toxicology for benzodiazepines post-mortem, and (3) “recent benzodiazepine and opioid exposure,” defined as those suicide deaths with recent benzodiazepine exposure who were also either prescribed an opioid within 30 days of death, or had positive toxicology post-mortem for opioids. Frequencies of demographic characteristics, mechanism of suicide, and other circumstances were assessed for total suicide deaths and these three subgroups.

## Results

Among the 3465 suicide deaths in Colorado between 2015 and 2017, 60% were linked to a record in the PDMP, as shown in Table 1. Linking to the PDMP meant that 60% of suicide deaths filled a prescription for a controlled substance during the 2014–2017 linkage timeframe. Among all 3465 suicide deaths, 31% had ever filled a benzodiazepine prescription preceding death (within the 2014–2017 linkage timeframe), according to the PDMP (Table 1). Additional information about the 3465 suicide deaths not shown in Table 1: Fourteen percent of the suicide deaths had filled a benzodiazepine prescription within 30 days before death, according to the PDMP. Approximately one-third (34%) had ever had benzodiazepine exposure. This exposure was defined as either ever filling a benzodiazepine prescription (within the linkage timeframe) as documented in the PDMP, or having positive toxicology post-mortem, per the Violent Death reporting.

**Table 1:** Colorado suicide deaths between 2015 and 2017, by PDMP linkage status, and benzodiazepine and opioid exposure status

	Total suicide deaths	Linked to PDMP record		Ever dispensed benzodiazepine^a^		Recent benzodiazepine exposure ^b^		Recent benzodiazepine and opioid exposure ^c^	
	Count	Count	Percent	Count	Percent	Count	Percent	Count	Percent
**Total**	3465	2070	59.7%	1058	30.5%	699	20.2%	331	9.6%
**Sex**									
**Female**	792	582	73.5%	373	47.1%	265	33.5%	143	18.1%
**Male**	2673	1488	55.7%	685	25.6%	434	16.2%	188	7.0%
**Age-Female**									
**0–14 yrs**	18	7	38.9%	*	*	*	*	*	*
**15–19 yrs**	64	29	45.3%	5	7.8%	6	9.4%	*	*
**20–24 yrs**	53	34	64.2%	16	30.2%	11	20.8%	8	15.1%
**25–34 yrs**	126	92	73.0%	60	47.6%	42	33.3%	21	16.7%
**35–44 yrs**	122	100	82.0%	70	57.4%	43	35.2%	19	15.6%
**45–54 yrs**	175	131	74.9%	88	50.3%	64	36.6%	32	18.3%
**55–64 yrs**	143	117	81.8%	84	58.7%	54	37.8%	35	24.5%
**65–74 yrs**	59	49	83.1%	37	62.7%	31	52.5%	18	30.5%
**75–84 yrs**	18	12	66.7%	5	27.8%	*	*	*	*
**85+ yrs**	14	11	78.6%	8	57.1%	7	50.0%	5	35.7%
**Age-Male**									
**0–14 yrs**	35	8	22.9%	*	*	*	*	*	*
**15–19 yrs**	150	46	30.7%	9	6.0%	11	7.3%	*	*
**20–24 yrs**	230	81	35.2%	32	13.9%	19	8.3%	8	3.5%
**25–34 yrs**	538	267	49.6%	117	21.7%	76	14.1%	25	4.6%
**35–44 yrs**	419	247	58.9%	125	29.8%	79	18.9%	38	9.1%
**45–54 yrs**	478	287	60.0%	138	28.9%	80	16.7%	28	5.9%
**55–64 yrs**	428	285	66.6%	157	36.7%	101	23.6%	49	11.4%
**65–74 yrs**	218	147	67.4%	63	28.9%	50	22.9%	30	13.8%
**75–84 yrs**	121	81	66.9%	30	24.8%	11	9.1%	*	*
**85+ yrs**	56	39	69.6%	14	25.0%	6	10.7%	*	*
**Suicide Mechanism**									
**Drugs/Overdose**	425	340	80.0%	247	58.1%	204	48.0%	147	34.6%
**Solids or Liquids**	18	9	50.0%	5	27.8%	*	*	*	*
**Gases and Vapors**	130	70	53.8%	33	25.4%	24	18.5%	9	6.9%
**Hanging/Asphyxiation**	956	536	56.1%	236	24.7%	126	13.2%	38	4.0%
**Firearm**	1743	998	57.3%	465	26.7%	294	16.9%	118	6.8%
**Jumping**	59	38	64.4%	22	37.3%	13	22.0%	7	11.9%
**Sharp Object**	45	22	48.9%	16	35.6%	10	22.2%	*	*
**Other/Unspecified**	89	57	64.0%	34	38.2%	24	27.0%	8	9.0%
**Other Circumstances**									
**No Known Mental Health Problem**	1489	723	48.6%	271	18.2%	170	11.4%	94	6.3%
**Known Mental Health Problem**	1976	1347	68.2%	787	39.8%	529	26.8%	237	12.0%
**No Known Physical Health Problem**	2171	1112	51.2%	519	23.9%	333	15.3%	115	5.3%
**Known Physical Health Problem**	1294	958	74.0%	539	41.7%	366	28.3%	216	16.7%
**No Known Alcohol or Substance Abuse Problem**	1979	1125	56.8%	552	27.9%	370	18.7%	174	8.8%
**Known Alcohol or Substance Abuse Problem**	1486	945	63.6%	506	34.1%	329	22.1%	157	10.6%

All percents are based on total suicide deaths in category

* Indicates fewer than three suicide deaths in category

^a “^Ever dispensed a benzodiazepine” is defined as having received a prescription for a benzodiazepine ever during the timeframe of the analysis (2014–2017)

^b^” Recent benzodiazepine exposure” is defined as benzodiazepines prescribed within 30 days of death or a positive toxicology for benzodiazepines post-mortem

^c^” Recent benzodiazepine and opioid exposure” is defined as those with recent benzodiazepine exposure who were also either prescribed an opioid within 30 days of death, or had positive toxicology post-mortem for opioids

We then assessed all 3465 suicide deaths for *recent* benzodiazepine exposure, which we defined as either receiving a prescription within 30 days of death or having positive toxicology results for benzodiazepines at death. One-fifth (20%) of all suicide deaths had recent benzodiazepine exposure (Table 1). Among suicide deaths with recent benzodiazepine exposure, nearly 50% also either filled an opioid prescription within 30 days of death, or had a positive toxicology post-mortem for opioids. Recent benzodiazepine exposure was more common among females than males (34% versus 16%), with percentages increasing among older female adults (Table 1). Among suicide deaths, those who died via drug overdose were more likely to have had recent benzodiazepine exposure (48%), compared to suicide deaths by firearm (17%), hanging/asphyxiation (13%) and all other methods (approximately 20%).

We examined additional information about the person’s circumstances and mental health issues at the time of death from the Colorado Violent Death Reporting System. Among all suicide deaths, 27% with a known/documented mental health issue had recent benzodiazepine exposure, while 11% had recent benzodiazepine exposure but no known or documented history of mental illness or treatment. Furthermore, 22% of suicide deaths with a known substance abuse or alcohol abuse problem had recent benzodiazepine exposure. Those with a known/documented physical health problem were also more likely to have had recent benzodiazepine exposure (28% versus 15%) or both recent benzodiazepine and opioid exposure (17% versus 5%).

## Discussion

We conducted a descriptive, public health assessment to determine whether benzodiazepine exposure was prevalent enough among Coloradans who died by suicide to warrant further investigation. This assessment indicated that almost a third of the persons who died by suicide during 2015–2017 had filled a benzodiazepine prescription during the 2014–2017 period or had positive toxicology post-mortem. Though persons had varying length of time between benzodiazepine prescription and the outcome of suicide death, 14% of the suicide deaths (almost half who ever filled a benzodiazepine prescription) had a prescription within 30 days of death. When recent benzodiazepine exposure included both benzodiazepine prescriptions within 30 days of death and/or post-mortem toxicology results indicating benzodiazepines, recent benzodiazepine exposure increased to 20% of the suicide deaths. Recent exposure was higher than 20% for females (34%) and persons who died via intentional drug overdose (48%). Recent benzodiazepine exposure was twice as common among persons with a known mental health problem (27%) than those without (11%). In addition, recent benzodiazepine exposure was higher among persons with known physical problems (28%) than those without (15%). These findings suggest that benzodiazepine exposure among suicide deaths is prevalent enough to warrant further investigation.

A national suicide study of 27 states participating in the National Violent Death Reporting System (including Colorado) found that 21% of suicide deaths from poisoning in 2015 had benzodiazepine as the class of substance causing death [4]. This prevalence increased to 25% for the subgroup with a current diagnosis of a mental health condition documented in coroner/medical examiner or law enforcement reports [4]. Our public health assessment suggests that recent *exposure* (not cause of death) among suicide deaths by drug poisoning (48%), which includes deaths with post-mortem toxicology results for benzodiazepine and a prescription within 30 days of death, was twice as prevalent as the 21% whose suicide death was caused by benzodiazepine in this national study.

The data in this assessment had many limitations. For example, we lacked information on reasons benzodiazepines were prescribed, whether prescriptions were taken as prescribed, or whether individuals abruptly stopped taking them or were tapering them on their own. We also lacked access to medical records, to assess the role of other medications or co-morbid conditions, including chronic pain. Due to small numbers, we did not assess if results varied for subpopulations defined by geography smaller than the state, such as for a county or city. In addition, we lacked access to an appropriate comparison group; hence, comparative risk could not be assessed. Given these limitations, our analysis was not meant to determine causality but to determine whether benzodiazepines and suicidality should be studied further from a population health perspective.

Strengths of this public health assessment are its novel use of the existing PDMP and the Violent Death Reporting System (both available in almost all states) and its novel definition of recent benzodiazepine exposure. Other states can quickly replicate this public health assessment. Future research can focus on the specific groups with recent benzodiazepine exposure identified in this analysis: females, persons who died from an intentional drug overdose, persons with a known mental health problem, and persons with known physical health problems. To address the limitations of this public health assessment, further research should be undertaken to determine linkages between benzodiazepine use and suicidal ideation, with assessments of medical/mental health records to identify and account for potential confounding factors (such as pre-existing conditions that lead to suicidal ideation). State health agencies that have access to more in-depth medical and mental health data should consider conducting analyses to examine associations between suicide deaths or attempts and benzodiazepine dosage, length of use, and tapering, while controlling for confounders. Prescribing patterns, by specialty and provider type, should also be examined, to determine whether prescribing/tapering guidelines, continuing education, academic detailing, informed consent, limitations on amounts prescribed and dispensed for certain acute conditions, or other public health policies should be established for benzodiazepines. Qualitative studies of prescribers and patients receiving benzodiazepine prescriptions could explain needs and barriers related to benzodiazepine use and use of alternative treatment, when warranted.

Both state and federal governments have policy levers that can impact prescribing. The use and potential for abuse of benzodiazepines has drawn the attention of state policy makers in the past. In 1989, amid fears of addiction to this family of drugs, New York State moved to regulate benzodiazepine prescriptions and enacted strict rules around prescribing [23]. A few states followed suit. A second wave of policy attention occurred in the late 1990s and first decade of the twenty-first century when family advocates became concerned about the over-prescription of these drugs for older adults and the sedating and amnesiac effects they could have. There was also concern that they increased the risks of falls, motor vehicle accidents, and other injuries. Policy makers responded by calling for informed consent procedures around prescribing and guidelines related to prescribing these medications for older adults [24]. Because of their deadly interaction with opioids, these drugs have again drawn attention from policy makers. To date, 15 states have enacted laws or promulgated regulations related to the co-prescribing of opioids and benzodiazepines. In addition, in 2016, the U.S. Food and Drug Administration added warnings to drug labeling regarding co-prescribing of opioids and benzodiazepines [25]. The goal of these policies was to reduce risks of addiction, accidental overdose, or dangerous interactions between drug classes [26].

No state or federal agencies have directly addressed the risk of suicidality that may be associated with benzodiazepines themselves. The idea that withdrawal or abrupt tapering may elevate risk of suicide is not broadly discussed by policy makers or public health officials. Further study, as described previously, should clarify the issue, and if warranted, policy levers should be identified to help combat the nation’s growing suicide crisis.

## Conclusion

Benzodiazepines have been linked to suicidal ideation. Yet population level assessments of benzodiazepine exposure among suicide deaths are rare. Our epidemiologic assessment indicates a relatively high prevalence of recent benzodiazepine exposure that warrants further investigation from both clinical and public health perspectives, especially among women, people with a mental health issue, and/or people with a physical health problem.

## Data Availability

All databases used in this study, including the PDMP and the Colorado Violent Death Reporting System, and Colorado’s Death Certificate Registry are authorized for public health use by Colorado law. Colorado state laws protect the confidentiality of the data utilized for this study; therefore, the authors cannot release identified data. For information on obtaining de-identified datasets, contact cdphe.healthstatistics@state.co.us or see https://www.datarequest.dphe.state.co.us/requests/create. The authors can respond to requests for summary information about the data linkage processes and analyses.

## Abbreviations

PDMP: Prescription Drug Monitoring Program
U.S.: United States
